# Elderly Patients at Risk of Malnutrition Presenting to a Tertiary Hospital in Nepal: A Descriptive Cross-sectional Study

**DOI:** 10.31729/jnma.8493

**Published:** 2024-03-31

**Authors:** Sabita Karki, Akriti Shree Dahal, Ramila Chaulagain, Sabitra Sapkota, Suman Chaudary

**Affiliations:** 1 National Academy of Medical Sciences, Bir Hospital, Mahankal, Kathmandu, Nepal; 2 Ministry of Health, Koshi Pradesh; 3 National Academy of Medical Sciences, Bir Hospital Nursing Campus, Mahankal, Kathmandu, Nepal; 4 National Academy of Medical Sciences, National Trauma Center, Mahankal, Kathmandu, Nepal

**Keywords:** *elderly*, *nutritional status*, *quality of life*

## Abstract

**Introduction:**

The nutritional status of elderly people is crucial for healthy aging, enabling them to maintain productive lives and reduce the progression of chronic diseases. Given that the quality of life tends to decline with age, it becomes particularly crucial for elderly individuals. Therefore, this study was aimed to find out the prevalence of elderly patients at risk of malnutrition in a tertiary hospital.

**Methods:**

This descriptive cross-sectional study was conducted among 281 elderly patients in a tertiary hospital from 2 January 2023 to 10 February 2023 after obtaining ethical approval. Nonprobability purposive sampling technique was used. A face-to-face interview was conducted using a structured interview schedule with the elderly in the absence of their caregiver using a standard Mini-Nutritional Assessment tool for nutritional status, and WHOQOL_OLD quality of life for the elderly to assess the quality of life. Point estimate at 95% Confidence Interval was calculated.

**Results:**

Out of 281 elderly patients enrolled in our study, 164 (58.36%) (52.64-64.16, 95% Confidence Interval) were at risk of malnutrition.

**Conclusions:**

The study concluded that older people could be at risk of malnutrition, which could impair their quality of life.

## INTRODUCTION

Globally, the population of the elderly is projected to double by 2050.^1^ The population census shows that the number of elderly increased from 1.5 million in 2001 to 1.95 million in 2021. Therefore, growing older is becoming a growing global concern.^2-4^ Nutritional requirements affecting nutritional status are complicated by a number of changes that come with aging.^5^ Even in developed countries, the prevalence of malnutrition varies. In Asia, the prevalence is 16-17%, with 10-24% in Nepal.^6-9^ Given that the quality of life is affected by age and nutrition addressing these issues is crucial.^10,11^

The Department of Health Services report only covers nutrition in children, girls, and pregnant women.^12^ The quality of life among the elderly is an important area of concern, reflecting the health status and well-being of this vulnerable population.

This study aimed to find out the prevalence of elderly patients at risk of malnutrition and their quality of life in a tertiary hospital.

## METHODS

A descriptive cross-sectional study was conducted in a tertiary hospital over a one-month period from 2 January 2023 to 10 February 2023. Ethical clearance was obtained from the Institutional Review Committee of the National Academy of Medical Sciences (Reference number: 146/2078/79). Data were collected from 281 elderly patients attending the geriatric and medical outpatient departments (OPD) after obtaining informed consent. The minimum sample size of the study was estimated with the following formula:


$$\begin{array}{ll} \text{n} & ={\text{Z}}^{2}\times \frac{\text{p}\times \text{q}}{{\text{e}}^{\text{2}}}\\ & =1.96^{2}\times \frac{0.65\times 0.35}{0.06^{2}}\\ & =243 \end{array}$$

Where,

n = minimum required sample size
Z = 1.96 at 95% Confidence Interval (CI)
p = prevalence of elderly at risk of malnutrition from a similar study, 65%^9^
q = 1-p
e = margin of error, 6%

The minimum required sample size was 243. However, a sample size of 281 was taken for the study. Nonprobability purposive sampling technique was used in the study. Every day, all elderly patients visiting the geriatric and medical OPD seeking treatment were identified from the OPD cards, especially in the medical OPD, as all age groups visited the medical OPD, and those aged 70 years and above visited the geriatric OPD. Those who were willing to participate and could respond to the interviewer were included in the study. The elderly patients who were unable to communicate were excluded from the study. Patients were taken to a separate room after their consultations in the OPD, and after that, written informed consent was obtained.

A face-to-face interview was conducted with the elderly in the absence of their caregiver using a standard Mini-Nutritional Assessment tool (MNA-SF) for nutritional status,^13^ and WHOQOL_OLD quality of life for elderly to assess the quality of life.^14^ MNA-SF is a validated tool developed by the Nestle Nutrition Institute. Both the English and Nepali versions of the tool were provided, and permission to use them was granted. The World Health Organization (WHO) also granted permission to use the WHOQOL_OLD quality of life tool. The English version of the tool was translated into Nepali, and back translation to English was done by language experts. The Cronbach's alpha for the tool was 0.72.

Descriptive statistics such as frequency, percentage, and mean were used to describe the quantitative variables. Nutritional status was categorised as follows: normal nutritional status (12-14 points), at risk of malnutrition (8-11 points), and malnourished (0-7 points) according to the tool. For quality of life, it was measured as either good or poor quality of life based on the mean value. A value greater than or equal to the mean was categorised as good quality of life, while a value below the mean was categorised as poor quality of life. Point estimate at 95% CI was calculated.

## RESULTS

Out of 281 elderly patients enrolled in our study, 164 (58.36%) (52.64-64.16, 95% CI) were at risk of malnutrition. The patients at risk of malnutrition were categorised based on their age, gender, marital status, educational status, types of family, smoking status, comorbidities, alcohol consumption and presence of adequate rest/sleep (Table 1).

**Table 1:** Demographic profile of elderly at risk of malnutrition (n= 164).

Variables	At risk of malnutrition n (%)
**Age**	
≤ 74	81 (49.39)
Greater than 74	83 (50.60)
**Gender**	
Male	73 (44.51)
Female	91 (55.48)
**Marital status**	
Married	93 (56.70)
Others	71 (43.29)
**Educational status**	
IIliterate	125 (76.21)
Literate	39 (23.78)
**Types of family**	
Nuclear	39 (23.78)
Joint	125 (76.21)
**Smoking**	
Yes	41 (25)
No	123 (75)
**Comorbidities**	
Yes	86 (52.43)
No	78 (47.56)
**Alcohol use**	
Yes	26 (15.85)
No	138 (84.14)
**Adequate rest/sleep**	
Yes	105 (64.02)
No	59 (35.97)

Among the elderly at risk of malnutrition, 102 (62.19%) had poor quality of life (Table 2).

**Table 2:** Quality of life of elderly atrisk of malnutrition (n = 164).

Quality of Life (Qol)	n (%)
Good Qol	62 (37.19)
Poor Qol	102 (62.19)

Out of a total score of 100 on various six domains of WHOQOL-Old, autonomy shows lowest scores that vary, with an average score of 11.43±3.13. Elderly show diversity in engagement in past, present, and future activities, with an average score of 12.06 (Table 3).

**Table 3:** Transformed score on various items of WHO_OLD QOL of elderly patient at risk of malnutrition (n = 164).

QOL_OLD Domain	Score	
	Minimum	Maximum
Sensory Abilities	4	20
Autonomy	4	19
Past, present and future activities	6	18
Social participation	5	18
Death and dying	4	20
Intimacy	4	20

## DISCUSSION

Malnutrition is a risk factor that is common among elderly but is often unreported. A number of factors put one at danger of malnutrition. The MNA tool is a quick and straightforward way to determine the nutritional status of the elderly. Early identification facilitates early nutritional treatment with nutritional therapy.^13^

In our study, only one-fourth of participants have a normal nutritional status, 15.7% are malnourished, and 58.36% are at risk of malnutrition. Similar findings were found in studies.^8,15,16^ Another study conducted in India too shows high prevalence of at risk of malnourished.^17^ However slight differences was observed in a study conducted in another hospital in Nepal.^7^ These differences might be due to disparities in lifestyle, healthcare facilities, and dietary habits among the elderly. Our study was conducted in one of the oldest government hospitals, where people from all over Nepal visited our centre.

More than half of the elderly have comorbidities, findings consistent with a study conducted in China.^18^ These findings have important clinical implications, suggesting that managing comorbidities may be crucial in improving or maintaining good nutritional status.

About two third of the elderly reported experiencing a poor quality of life, and our findings align with prior research in similar populations.^11,19,20^ This highlights the severity of the issues and suggests efforts to address these issues.

Likewise, in the item-wise domain analysis, lower scores were observed in all domains compared to another study.^11,20^ The contrasting findings may be due to the presence of specific health conditions or comorbidities within our elders, which may affect the quality of life scores. Individuals with chronic illnesses or disabilities may report lower scores compared to a healthier population. In item wise domain, particularly autonomy shows the lowest score among all. The lowest mean suggests a potential area of concern regarding autonomy. Similarly, past present and future activities are followed by autonomy in lowest scores. The variation in individual scores highlight the diversity of experiences within the population.

This study finding is not representative of the general population as the study was conducted in a selective tertiary hospital setting. Elderly may over report or underreport particular elements of their habits, nutritional status, and quality of life, which can lead to biases when self-reported data is used to gather the information.

## CONCLUSIONS

Our study investigated the prevalence of malnutrition risk and quality of life which revealed a noteworthy number at risk of malnutrition, emphasising a significant concern. Factors which might be influencing nutritional status included age, comorbidities, and lifestyle behaviours. A substantial proportion of participants reported poor quality of life, emphasising the need for targeted interventions to address the health challenges faced by the elderly population. Addressing not only nutritional aspects but also promoting healthy lifestyles to manage comorbidities are crucial components of comprehensive health strategies to improve the overall quality of life.
